# Eosinophils as predictive biomarkers in anti-programmed cell death 1 monotherapy for non-small cell lung cancer

**DOI:** 10.3389/fimmu.2025.1574314

**Published:** 2025-10-07

**Authors:** Takahiro Uchida, Kazuyuki Nakagome, Kosuke Hashimoto, Hidetoshi Iemura, Yuki Shiko, Atsuto Mouri, Ou Yamaguchi, Yoshitaka Uchida, Yoshiaki Nagai, Tomoyuki Soma, Kyoichi Kaira, Makoto Nagata, Hiroshi Kagamu

**Affiliations:** ^1^ Department of Respiratory Medicine and Allergy Center, Saitama Medical University, Saitama, Japan; ^2^ Department of Respiratory Medicine, Saitama Medical University International Medical Center, Saitama, Japan; ^3^ Department of Biostatistics, Graduate School of Medicine, Saitama Medical University, Saitama, Japan

**Keywords:** anti-PD-1 monotherapy, eosinophils, immune checkpoint inhibitor, lung cancer, T- lymphocyte subset

## Abstract

**Background:**

The relationship between eosinophilia and cancer development has recently been investigated. However, the role of eosinophils in tumor immunity, particularly in the context of immune checkpoint inhibitor (ICI) therapy, remains poorly understood.

**Methods:**

We investigated the relationship between peripheral blood eosinophil and T-lymphocyte subsets and the clinical characteristics of patients undergoing anti-programmed cell death-1 (PD-1) monotherapy for non-small cell lung cancer (NSCLC). The study included 204 patients treated with nivolumab monotherapy, and clinical data and treatment responses were recorded. PBMCs were collected from 44 out of 204 patients before treatment to analyze T-lymphocyte subsets, focusing on their correlation with blood eosinophils.

**Results:**

The percentage of blood eosinophils before nivolumab treatment was positively correlated with the percentage of effector memory subsets in both CD4^+^ (r = 0.43, p = 0.0045) and CD8^+^ T cells (r = 0.35, p = 0.020). It was negatively correlated with the percentage of naïve subsets of CD4^+^ T cells and positively correlated with the percentage of inducible T cell co-stimulator cells among CD8^+^ T cells. Patients with higher eosinophil levels (≥1.7%) before nivolumab treatment exhibited significantly longer progression-free survival (log-rank p = 0.014) and overall survival (log-rank p = 0.001) than those with lower eosinophil levels. An early increase in the eosinophil count after treatment was also associated with a better response to nivolumab.

**Conclusion:**

Higher blood eosinophil levels may indicate activated T-cell immunity and may be a promising biomarker for the efficacy of anti-PD-1 monotherapy in patients with NSCLC.

## 1 Introduction

Eosinophils contribute to the pathogenesis of allergic diseases such as bronchial asthma by releasing specific granules (e.g., major basic protein (MBP)), lipid mediators, and reactive oxygen species (1). Furthermore, recent studies suggest that they release extracellular DNA traps during eosinophil extracellular trap cell death, which can exacerbate inflammation (2). Eosinophils play an important role in asthma development. Anti-IL-5 treatment reduces blood eosinophil counts and the frequency of exacerbations in eosinophil-dominant severe asthma (3, 4). However, it has also been suggested that eosinophils have multiple functions, including the maintenance of homeostasis (5–8). Eosinophils can improve insulin resistance, enhance glucose tolerance, and are involved in metabolic homeostasis through the maintenance of adipose alternatively activated macrophages (8). Furthermore, phenotypic diversity in eosinophils has drawn attention, particularly in mice (9, 10). Therefore, eosinophils may play different roles depending on the location and situation in which they work.

Lung cancer is a malignant disease with a poor prognosis (11–15). Platinum-based chemotherapy has been the standard of care for patients with advanced lung cancer and can prolong survival by only a few months (16–18). Immune checkpoint inhibitors (ICIs) have changed the treatment paradigm for cancer patients. ICIs in clinical use include antagonistic antibodies (Abs) that block cytotoxic T-lymphocyte-associated antigen 4 (CTLA-4), programmed cell death-1 (PD-1), and programmed cell death ligand-1 (PD-L1). Targeting these immune checkpoint molecules in patients with cancer has resulted in durable responses (18, 19). Approximately 15% of patients with advanced non-small cell lung cancer (NSCLC) survive for more than five years on anti-PD-1 monotherapy (18).

However, the role of eosinophils in cancer development remains controversial (20–26). The anti-tumor role of eosinophils has been reported in several types of cancer (20–23). In contrast, the pro-tumoral role of eosinophils has been suggested in several cancers, including lung adenocarcinoma (20, 21, 24–26). Recently, the association between blood eosinophil count and the efficacy of ICI therapy has been highlighted (27–31). In patients with malignant lymphoma treated with ICI therapy, some studies indicate that high pretreatment blood eosinophil counts or early increases in eosinophil count after treatment are associated with an improved clinical response (27, 28). Furthermore, this association has also been explored in NSCLC, and similar findings have been suggested in some studies (29–31). However, further studies are necessary to implement these parameters in clinical use, and the underlying immunological mechanisms remain unclear.

Against this background, we examined the relationship between peripheral blood eosinophils and clinical characteristics including treatment responses in nivolumab-treated NSCLC patients. We also analyzed CD4^+^/CD8^+^ naïve, central-memory, and effector-memory T-cell subsets in some patients, as effector CD4^+^ and CD8^+^ T cells play important roles in the efficacy of ICI treatment (32–37).

## 2 Methods

### 2.1 Study design

Two hundred twenty-six consecutive patients with advanced NSCLC who received nivolumab (anti-PD-1 Ab) monotherapy at Saitama Medical University International Medical Center between February 2016 and March 2019 were enrolled. Patients were eligible if (i) a complete blood count including baseline eosinophil count was available within 14 days before the first nivolumab dose; (ii) they had received nivolumab as second- or later-line therapy and completed at least one treatment cycle; and (iii) they had not received ICIs in any previous line of treatment. Patients lacking mandatory baseline clinical or laboratory data were excluded. Clinicopathological characteristics and subsequent treatments were extracted from electronic medical records. During the study period, nivolumab was approved in Japan only as second-line or subsequent treatment option. We therefore restricted enrolment to patients who received the drug as second-line or later treatment. All procedures followed the ethical standards of the Institutional Review Board of Saitama Medical University International Medical Center, and the 1964 Declaration of Helsinki and its subsequent amendments. Written informed consent was obtained from all patients prior to sample collection. The Institutional Review Board of Saitama Medical University International Medical Center approved this study (approval number: 15-221).

### 2.2 Treatment

Nivolumab was administered intravenously at 3 mg/kg or 240 mg/day every 2 weeks or 480 mg/day every 4 weeks.

### 2.3 Assessment of clinical data

Before nivolumab monotherapy, baseline tumor and response assessments were performed based on computed tomography (CT), magnetic resonance imaging (MRI), or positron emission tomography (PET)-CT findings, according to local standards. The trial used Response Criteria in Solid Tumors (RECIST) version 1.1. Progression-free survival (PFS) was recorded as the time from the initiation of nivolumab therapy to the confirmation of disease progression or death from any cause. The PFS was censored on the day of the last follow-up examination in patients who were free of progression. OS was defined as the time from the initiation of nivolumab monotherapy to death from any cause (event) or the last contact (censored). Routine physical examinations, laboratory tests, and imaging were performed to assess the safety at each follow-up visit. Allergic diseases were defined as bronchial asthma, allergic rhinitis, conjunctivitis, or atopic dermatitis. The numbers and percentages of eosinophils, neutrophils, lymphocytes, and basophils were measured simultaneously as part of the complete blood count before and during nivolumab monotherapy. The highest eosinophil count and percentage during the first two months of nivolumab treatment without corticosteroids were defined as eosinophil max2m and eosinophil max2m%, respectively.

### 2.4 Analysis of PBMC

PBMCs were analyzed in accordance with the methodology described in our previous study (23), involving the use of the same mAbs and a BD LSR Fortessa flow cytometer (Becton, Dickinson and Company) for cell staining. Briefly, blood specimens were collected from Becton Dickinson Vacutainer Systems into heparin-coated CPT Vacutainer tubes. The cells were centrifuged at 1500 × *g* for 20 min at ambient temperature to isolate PBMCs using a Ficoll density gradient. The isolated PBMCs were then preserved at –80 °C in Cellbanker2 (Nippon Zenyaku Kogyo Co., Ltd.) and stored in liquid nitrogen for seven days. T cell subsets were cultivated in RPMI1640 medium containing 10% fetal calf serum (FCS) for 32–48 h before staining. One million cells were subjected to direct immunofluorescence staining with mAbs conjugated with fluorescent dyes to evaluate the cell surface markers. Cells were treated with Abs in 100 μL FACS buffer, which consisted of PBS with 5% FCS, for 30 min at 4 °C. A dual wash was performed using 1 mL FACS buffer. Intracellular staining preparations were conducted with a FoxP3 fixation and permeabilization kit according to eBioscience’s guidelines at 4 °C. After two washes in FACS buffer, cells were fixed in 0.5% paraformaldehyde in PBS. A total of 10,000 cells from each sample were analyzed using an LSR Fortessa microfluorometer (Becton Dickinson), and the data were processed using the FlowJo software program. Furthermore, we characterised peripheral CD4^+^ and CD8^+^ T-cell subsets because the distribution of naïve, central-memory, and effector-memory compartments is regarded as a functional indicator of immune competence during PD-1 blockade.

### 2.5 Statistical analysis

Statistical significance was set at P < 0.05. The Cochran–Armitage trend test, Mann–Whitney U test, Spearman’s rank correlation, chi-square test, Fisher’s exact test, Kruskal–Wallis test, and Steel’s multiple comparisons test were used to analyze the associations between eosinophil levels and clinical characteristics, as appropriate. Receiver operating characteristic (ROC) curves and logistic regression analyses were used to investigate the use of eosinophils as therapeutic biomarkers. The log-rank test and Cox proportional hazards models were used to examine the relationship between the PFS and OS. All analyses were performed using JMP 10 (SAS Institute, Cary, North, USA).

## 3 Results

### 3.1 Patient selection and characteristics

**Figure 1:**
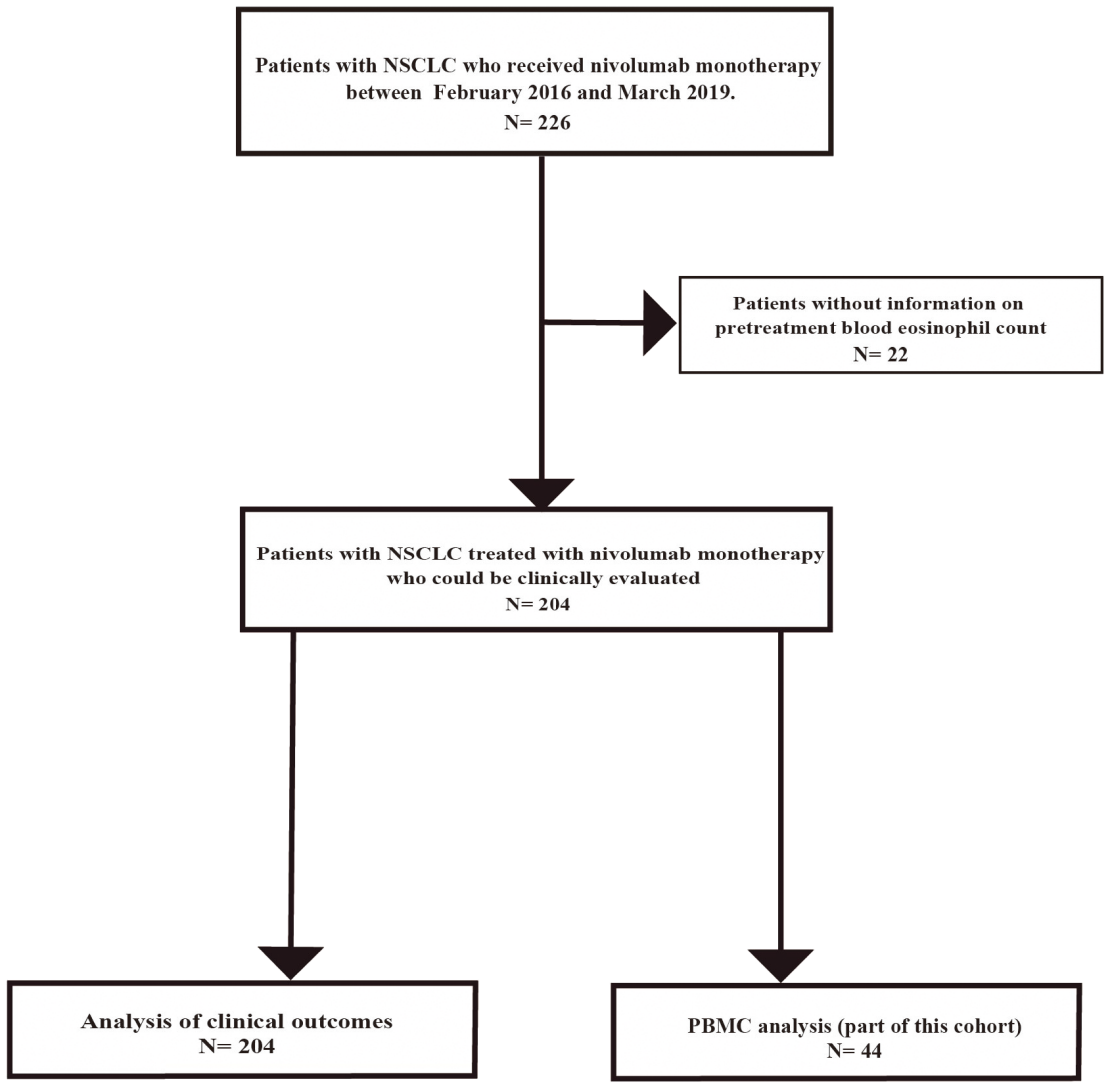
Flow diagram of patient selection and exclusion. Flowchart of the study involving 226 patients with advanced NSCLC who received nivolumab monotherapy at Saitama Medical University International Medical Center between February 2016 and March 2019. Patients with missing data, including pretreatment blood eosinophil count, were excluded. Finally, 204 patients were included in this study. PBMCs were obtained from 44 out of 204 patients before nivolumab monotherapy after additional consent for peripheral blood sampling.

Between February 2016 and March 2019, 226 consecutive patients with advanced or recurrent NSCLC were screened for eligibility (**Figure 1**). Twenty-two patients were excluded owing to missing baseline data, 18 patients lacked a pretreatment eosinophil count, and 4 patients had incomplete clinical records. Accordingly, 204 patients met all inclusion criteria and were included in the final analysis (**Figure 1**). Among 204 patients, PBMCs were obtained from 44 patients before nivolumab monotherapy after additional consent for peripheral blood sampling (**Figure 1**).

The patient characteristics are shown in **Table 1**. The median age was 69 years (range, 31–89 years). The study included 148 men (72.5%) and 56 women (27.5%). All patients received nivolumab as a second-line or later therapy. The Eastern Cooperative Oncology Group performance status score was 0–1 in 172 (84.3%) patients. A history of smoking was observed in 158 (77.5%) patients. Allergic diseases were present in 68 patients (33.3%), and 9 patients (4.4%) used inhaled corticosteroids. Adenocarcinoma was present in 120 patients (58.8%). Forty-three patients (21.1%) experienced recurrence after surgery or radiotherapy, and 37 (18.1%) and 124 (60.8%) patients had stage III and IV tumors, respectively. This study included 38 patients (18.6%) with driver mutations, 37 (18.1%) with epidermal growth factor receptor (EGFR) driver mutations, and one (0.5%) with anaplastic lymphoma kinase (ALK) driver mutations. The tumor proportion score (TPS) was assessed using PD-L1 immunohistochemistry (clone 22C3) in 48 patients (23.5%). The TPS was 1–49% in 19 patients (9.3%) and ≥50% in 5 patients (2.5%).

**Table 1:** Patient characteristics.

Variables	All patients n＝204
Age (median)	69 (31-89)
Sex Male/Female	148/56 (72.5%/27.5%)
Line of therapy (≥2nd line) 2nd line/3rd line/≥4th line	137/29/38 (67.2%/14.2%/18.6%)
PS 0-1/≥2	172/32 (84.3%/15.7%)
Smoking history Former/Never	158/46 (77.5%/22.5%)
Allergic disease +/-	68/136 (33.3%/66.7%)
Inhaled corticosteroids Yes/No	9/195 (4.4%/95.6%)
Histology Adeno/Squamous/Others	120/52/32 (58.8%/25.5%/15.7%)
Disease stage III/IV/Recurrence	37/124/43 (18.1%/60.8%/21.1%)
Mutation status Wild type/EGFR/ALK	166/37/1 (81.4%/18.1%/0.5%)
PD-L1 (TPS) <1%/1-49%/≥50%/Unknown	24/19/5/156 (11.8%/9.3%/2.5%/76.5%)

PS, performance status; Adeno, adenocarcinoma; Squamous, squamous cell carcinoma; recurrence, recurrence after surgical resection; EGFR, epidermal growth factor receptor; ALK, anaplastic lymphoma kinase; PD-L1, programmed death ligand-1; TPS, tumor proportion score.

### 3.2 Change in blood eosinophil levels after nivolumab administration

**Figure 2:**
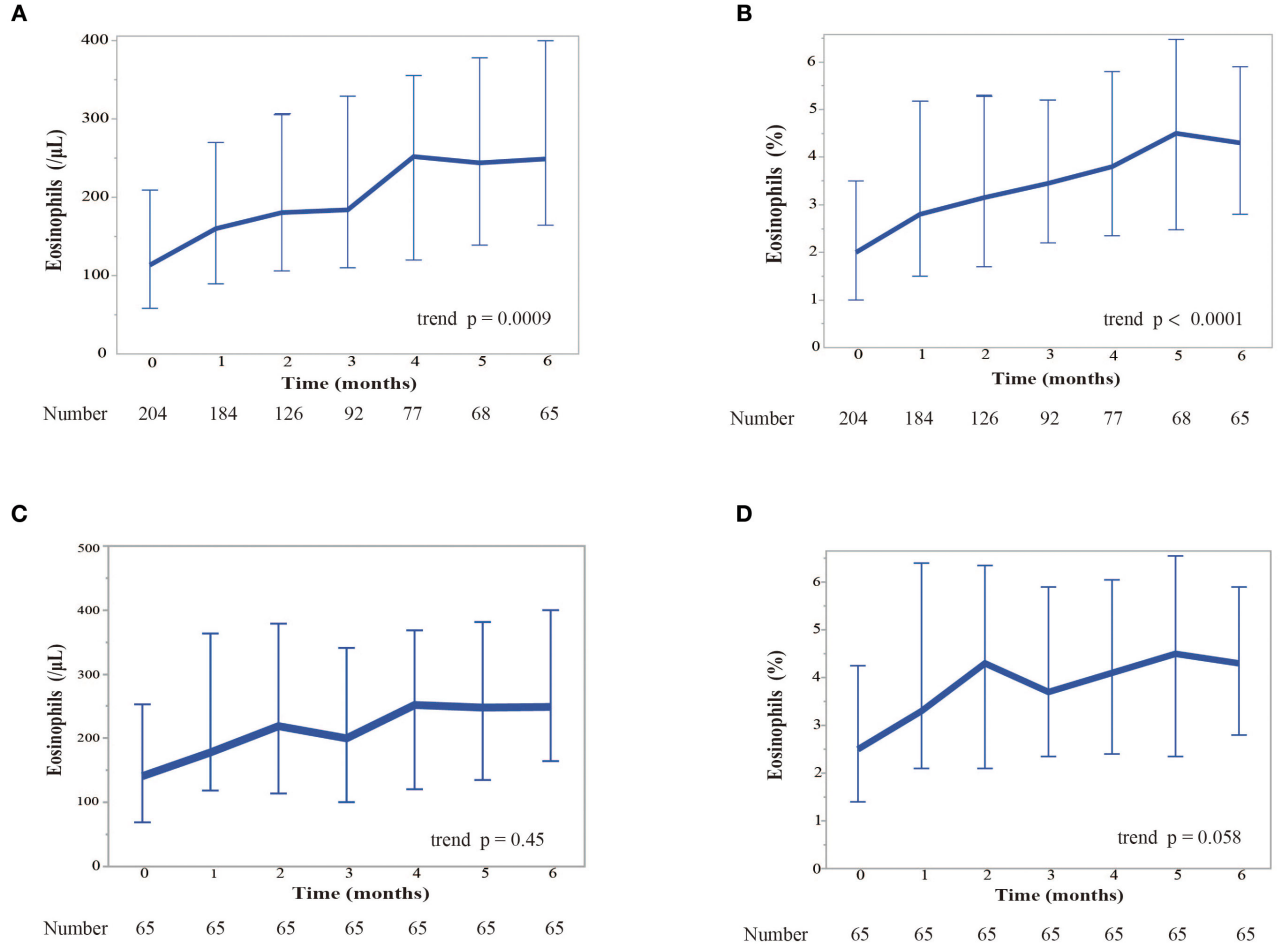
Kinetics of the response of eosinophil levels to nivolumab monotherapy over 6 months. **(A)** Kinetics of blood eosinophil counts (in cells per microliter). We examined the change of blood eosinophil counts monthly for 6 months while patients were treated with nivolumab. Patients who required corticosteroids due to immure-related adverse events and those with PD were excluded. Eosinophil counts are shown as median values. Error bars represent the interquartile range. The Cochran-Armitage trend test was used for the analysis. A significant increase in the eosinophil count was observed over time (trend p = 0.0009). **(B)** Kinetics of the median percentage of eosinophils to white blood cells. Eosinophil percentages are shown as median values. Error bars represent the interquartile range. The Cochran-Armitage trend test was used for the analysis. A significant increase in the relative percentage of eosinophils was observed (trend p < 0.0001). **(C)** Kinetics of blood eosinophil counts restricted to the patients who completed 6 months of nivolumab treatment. **(D)** Kinetics of the median eosinophil percentage restricted to the patients who completed 6 months of nivolumab treatment.

**Figure 3:**
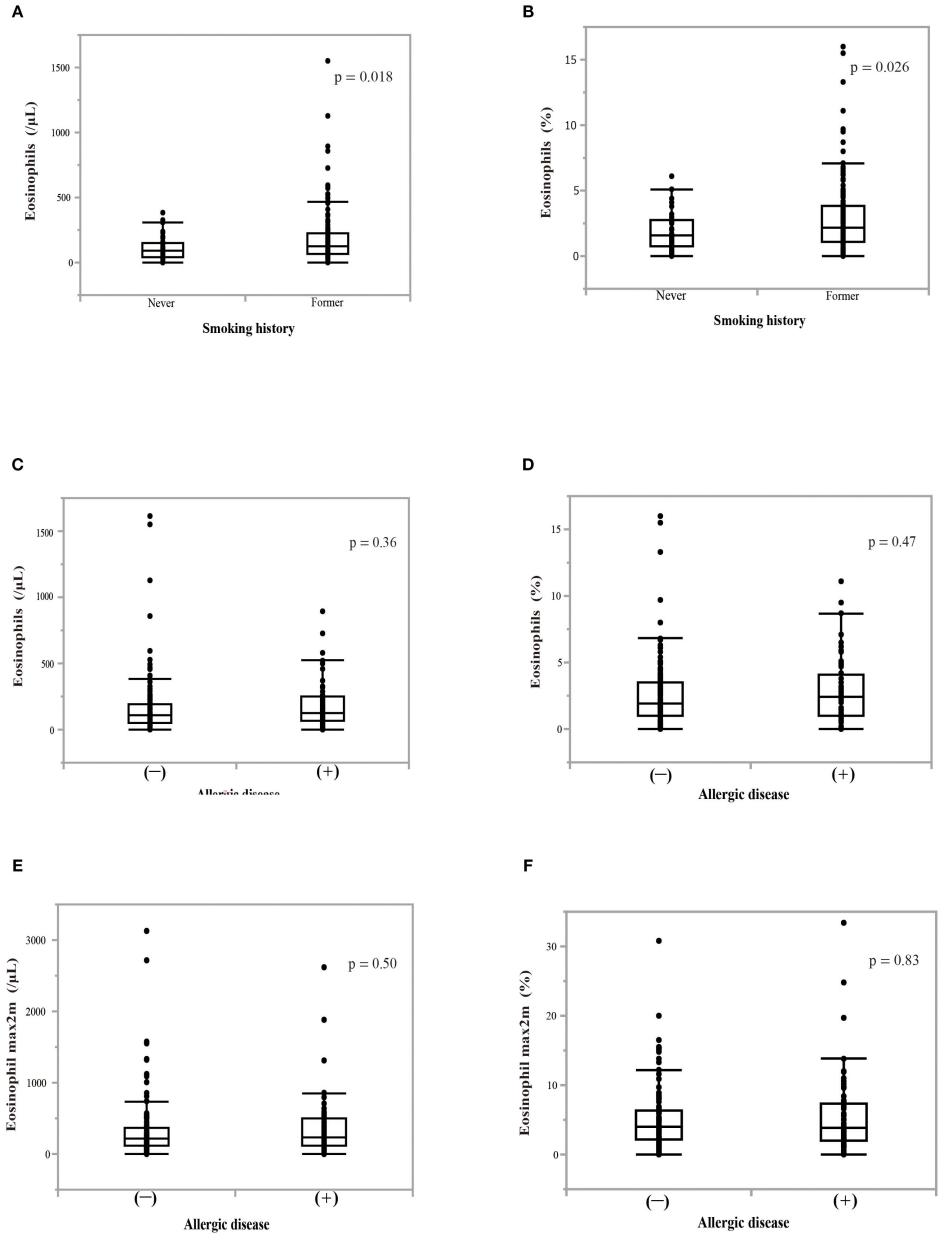
Effect of smoking history or allergic disease on blood eosinophil counts or percentages. **(A)** The effect of a smoking history on pretreatment blood eosinophil counts. **(B)** The effect of a smoking history on pretreatment percentage of blood eosinophils to white blood cells. P-values indicate the significance of differences between groups. The Mann–Whitney U test was utilized for analyses. **(C)** The effect of the presence of allergic disease on pretreatment blood eosinophil counts. **(D)** The effect of the presence of allergic disease on pretreatment blood eosinophil percentage. **(E)** The effect of the presence of allergic disease on the highest eosinophil counts over a 2-month period (eosinophil max2m). **(F)** The effect of the presence of allergic disease on the highest eosinophil percentage over a 2-month period (eosinophil max2m%).

We examined the change in blood eosinophil counts every month for 6 months while patients were treated with nivolumab monotherapy (**Figure 2**). Patients who needed corticosteroid treatment due to immune-related adverse events and those who were classified as having progressive disease (PD) according to RECIST version 1.1, were excluded from the analyses. Nivolumab monotherapy induced a significant increase in both the blood eosinophil count (p = 0.0009) and in the percentage of eosinophils to white blood cells (p < 0.0001) in all treated patients (**Figures 2A, B**). When analyzed in patients who completed 6 months of nivolumab treatment (n = 65), it induced a slight, but not significant, increase in the blood eosinophil percentage (**Figures 2C, D**). In patients with a history of smoking, the pretreatment blood eosinophil count (p = 0.018) and eosinophil percentage (p = 0.026) were higher than those in patients without a history of smoking (**Figures 3A, B**). However, the highest eosinophil count and percentage during the first 2 months of nivolumab monotherapy did not differ between patients with and without a history of smoking (data not shown). The presence of allergic diseases did not affect the pretreatment blood eosinophil count, eosinophil percentage, or the highest eosinophil count and eosinophil percentage during the first two months of nivolumab monotherapy (eosinophil max2m and eosinophil max2m%, respectively) (**Figures 3C–F**). No correlation was observed between TPS and eosinophil count or percentage (data not shown).

### 3.3 Association between pretreatment eosinophil levels and T cell subpopulations

**Figure 4:**
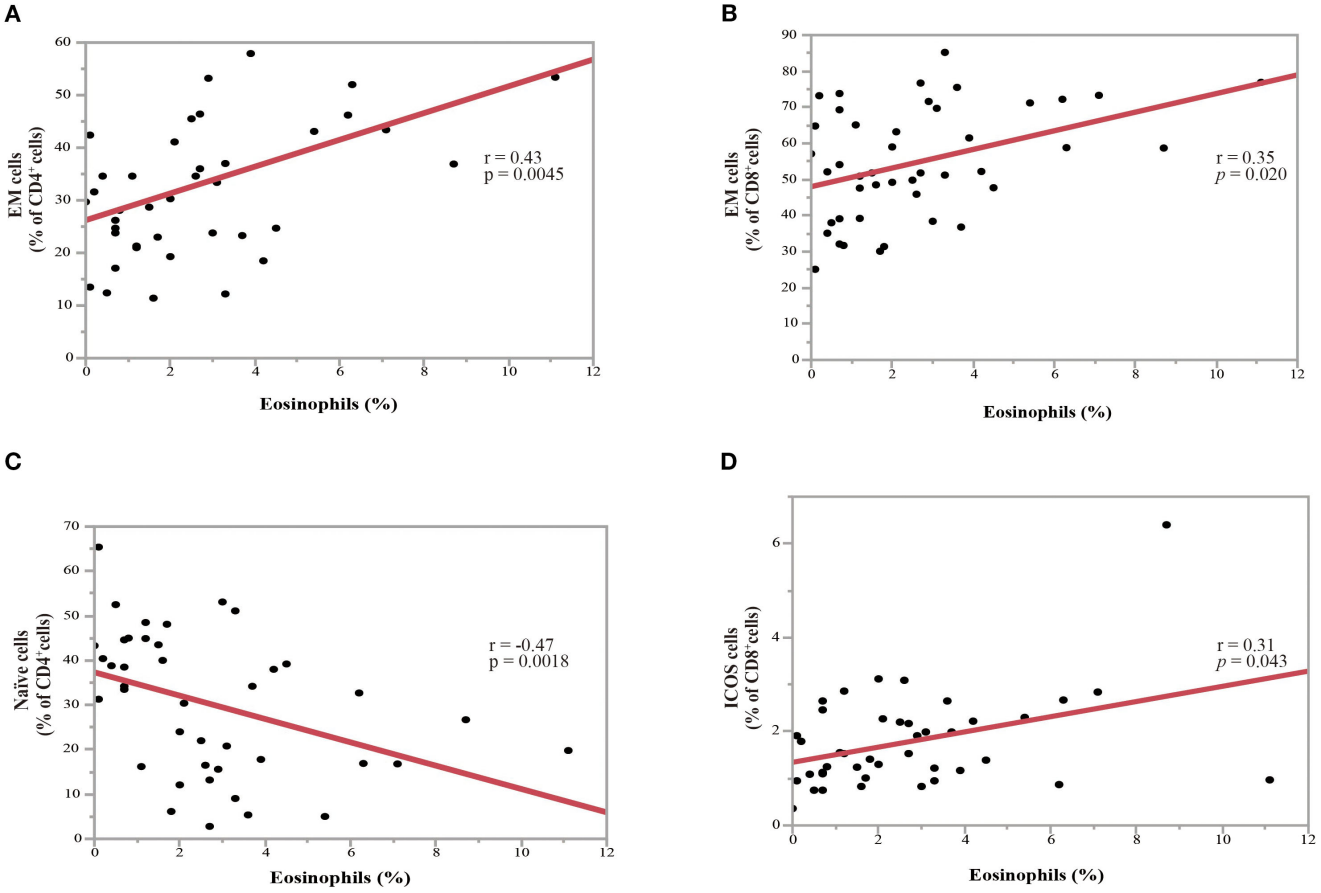
Association between the percentage of pretreatment blood eosinophils and the percentage of T-cell subpopulations in NSCLC patients before nivolumab treatment. **(A)** Correlation between the percentages of eosinophils and the effector memory (EM) subset in CD4^+^ T-cells (correlation coefficient =0.43, p = 0.0045). **(B)** Correlation between the percentages of eosinophils and the EM subset in CD8^+^ T-cells (correlation coefficient = 0.35, p = 0.020). **(C)** Correlation between the percentages of eosinophils and the naïve subset in CD4^+^ T-cells (correlation coefficient = -0.47, p = 0.0018). **(D)** Correlation between the percentages of eosinophils and inducible T-cell co-stimulator^+^(ICOS^+^) cells in CD8^+^ T-cells (correlation coefficient = 0.31, p = 0.043). Each plot represents individual patient data (black dots), with the red line indicating the linear regression fit. Statistical significance (p-values) and correlation coefficients (r-values) are displayed for each correlation. Spearman’s rank correlation coefficient was used to evaluate bivariate correlation.

**Figure 5:**
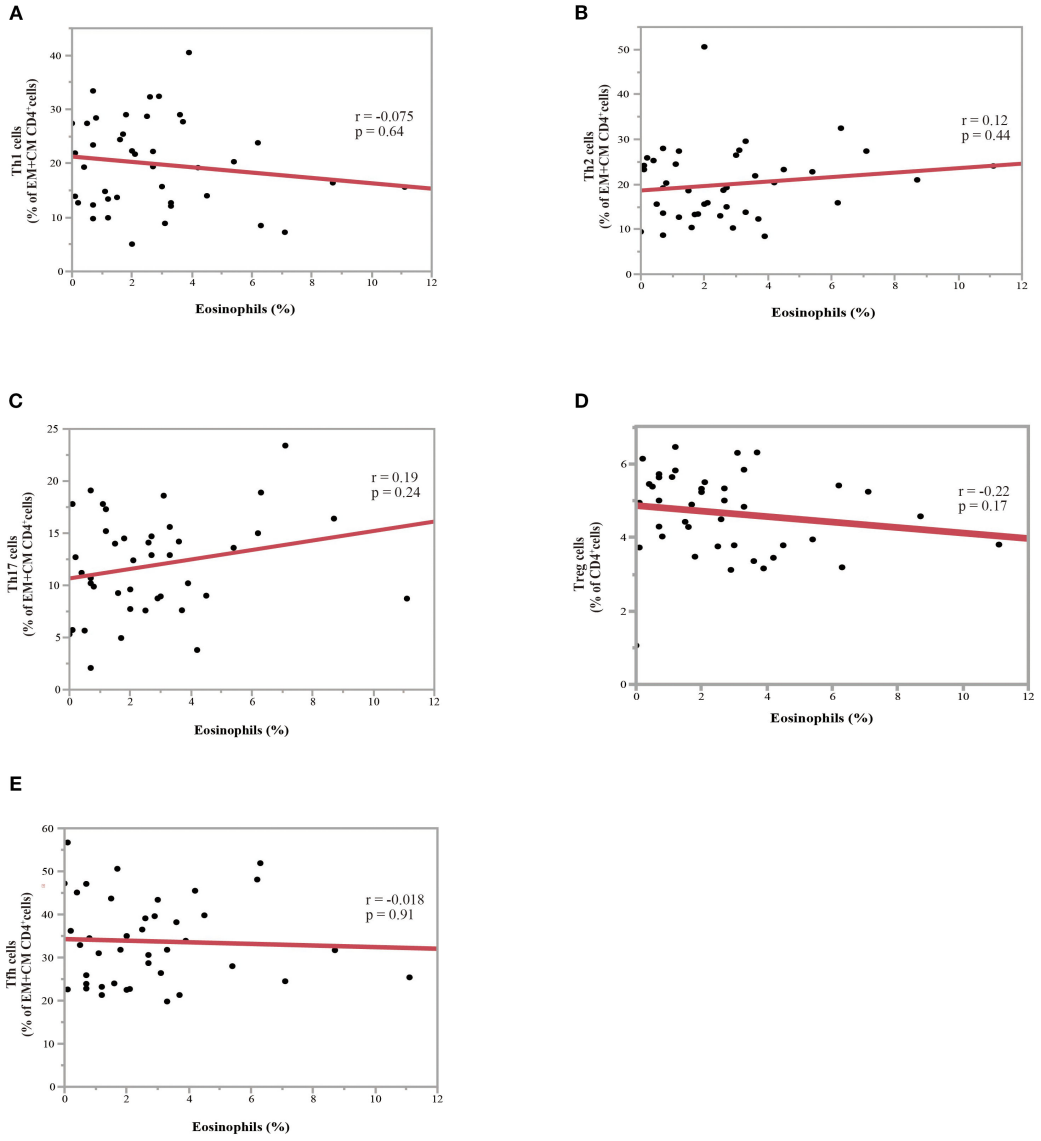
Association between eosinophil percentage and Th subsets in NSCLC patients before nivolumab treatment. **(A)** Correlation between the percentage of eosinophils and the ratio of Th1 to effector memory (EM) plus central memory (CM) CD4^+^ T-cells. **(B)** Correlation between the eosinophil percentage and the ratio of Th2 to EM plus CM CD4^+^ T-cells **(C)** Correlation between the eosinophil percentage and the ratio of Th17 to EM plus CM CD4^+^ T-cells. **(D)** Correlation between the eosinophil percentage and the ratio of Treg to CD4^+^ T-cells. **(E)** Correlation between the eosinophil percentage and ratio of Tfh to EM plus CM CD4^+^ T-cells. Each plot represents individual patient data (black dots), with the red line indicating the linear regression fit. Statistical significance (p-values) and correlation coefficients (r-values) are displayed for each correlation. Spearman’s rank correlation coefficient was used to evaluate bivariate correlation.

A PBMC analysis was performed in 44 out of 204 patients before the initiation of nivolumab monotherapy. Baseline characteristics of the patients whose PBMCs were analyzed were summarized in **Supplementary Table 1** and comparable to those of the overall cohort. Th cell polarity was classified using chemokine receptor expression patterns, which classified CXCR3^+^CCR4^-^CCR6^-^ as Th1 type, CXCR3^-^CCR4^+^CCR6^-^ as Th2 type, CXCR3^-^CCR4^+^CCR6^+^ as Th17 type, CXCR5^+^ as T follicular helper (Tfh) type, and FoxP3^+^CD45RA^-^ as Treg type. Memory cells are classified into three types: CCR7^+^CD45RA^-^central memory (CM), CCR7^-^CD45RA^-^ effector memory (EM), and CCR7^-^CD45RA^+^ effector memory re-expressing CD45R (EMRA) cells. **Figure 4** illustrates the relationship between eosinophil percentage and various memory cell subpopulations. The findings indicated a positive but moderate correlation between eosinophil percentage and CD4^+^ and/or CD8^+^ percentage, specifically for the percentage of the EM subset in CD4^+^ T cells (**Figure 4A**; correlation coefficient = 0.43, p = 0.0045) and EM subset in CD8^+^ T cells (**Figure 4B**; correlation coefficient = 0.35, p = 0.020). Conversely, a negative correlation was identified between eosinophil percentage and the percentage of naïve CD4^+^ T cell subsets (**Figure 4C**; correlation coefficient = -0.47, p = 0.0018). The percentage of inducible T-cell co-stimulator (ICOS)-positive CD8^+^ T cells was also moderately correlated with the eosinophil percentage (correlation coefficient = 0.31, p = 0.043) (**Figure 4D**). However, the eosinophil percentage was not correlated with the percentage of Th1, Th2, Th17, Treg, and Tfh cells (**Figure 5**), suggesting that the presence of eosinophils was not associated with the polarity of Th cells.

### 3.4 Peripheral eosinophils as predictors of the therapeutic response to nivolumab monotherapy

**Figure 6:**
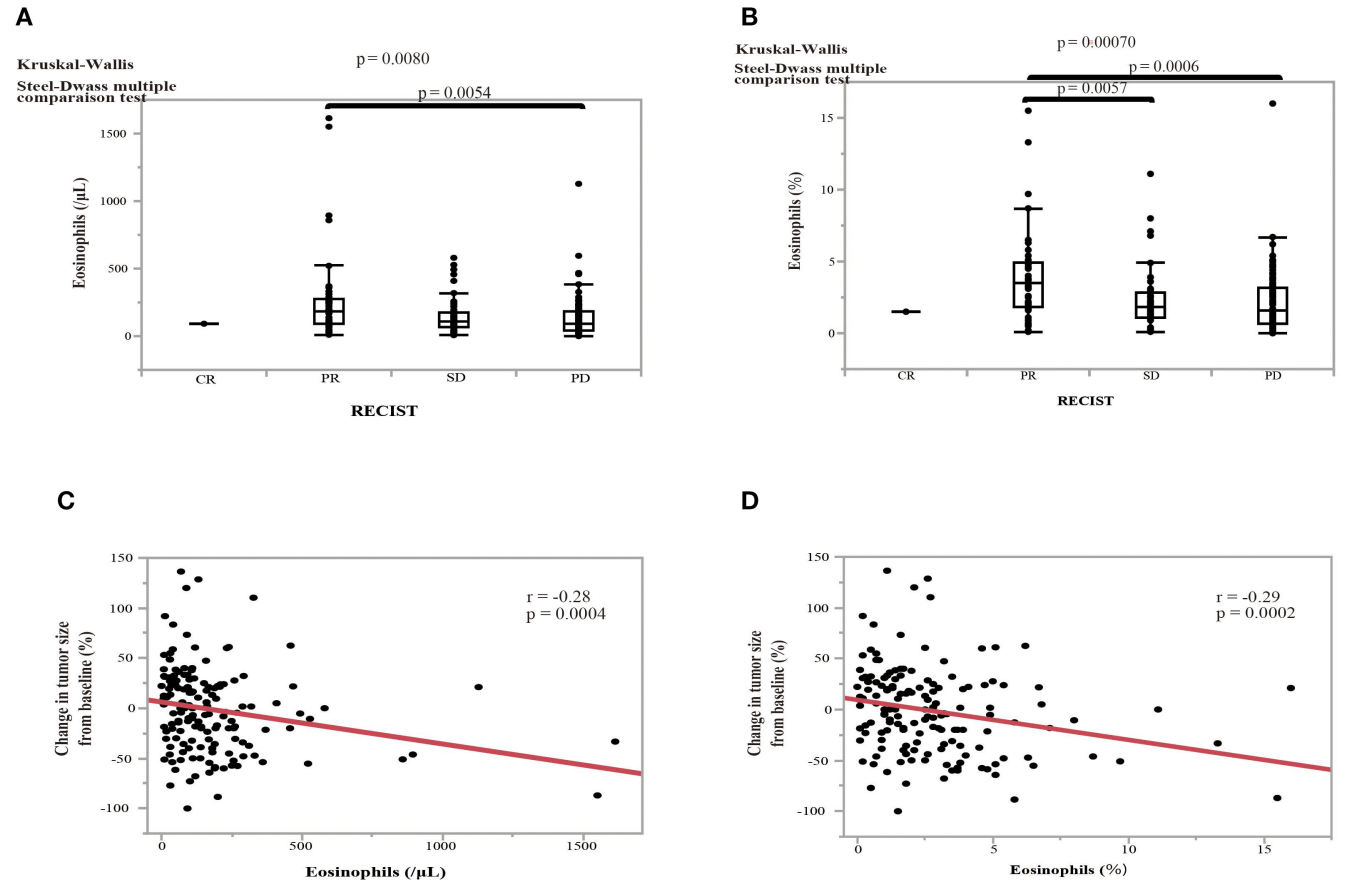
Effect of pretreatment eosinophil levels on the treatment response to nivolumab and the association between pretreatment eosinophil levels and the change in tumor size in NSCLC patients. **(A)** The effect of pretreatment eosinophil counts on the treatment response according to the RECIST criteria: complete response (CR); partial response (PR), stable disease (SD), and progressive disease (PD). **(B)** The effect of the pretreatment eosinophil percentage on the treatment response. Statistical significance, as determined by Kruskal–Wallis and Steel–Dwass multiple comparison tests. Error bars in boxplots represent the interquartile range. **(C)** The relationship between the pretreatment eosinophil count and the change in tumor size from baseline. **(D)** The relationship between the eosinophil percentage and the change in tumor size from baseline. Red line indicates the linear regression fit. P-values indicate the level of statistical significance. Spearman’s rank correlation coefficient was used to evaluate bivariate correlation.

**Figure 7:**
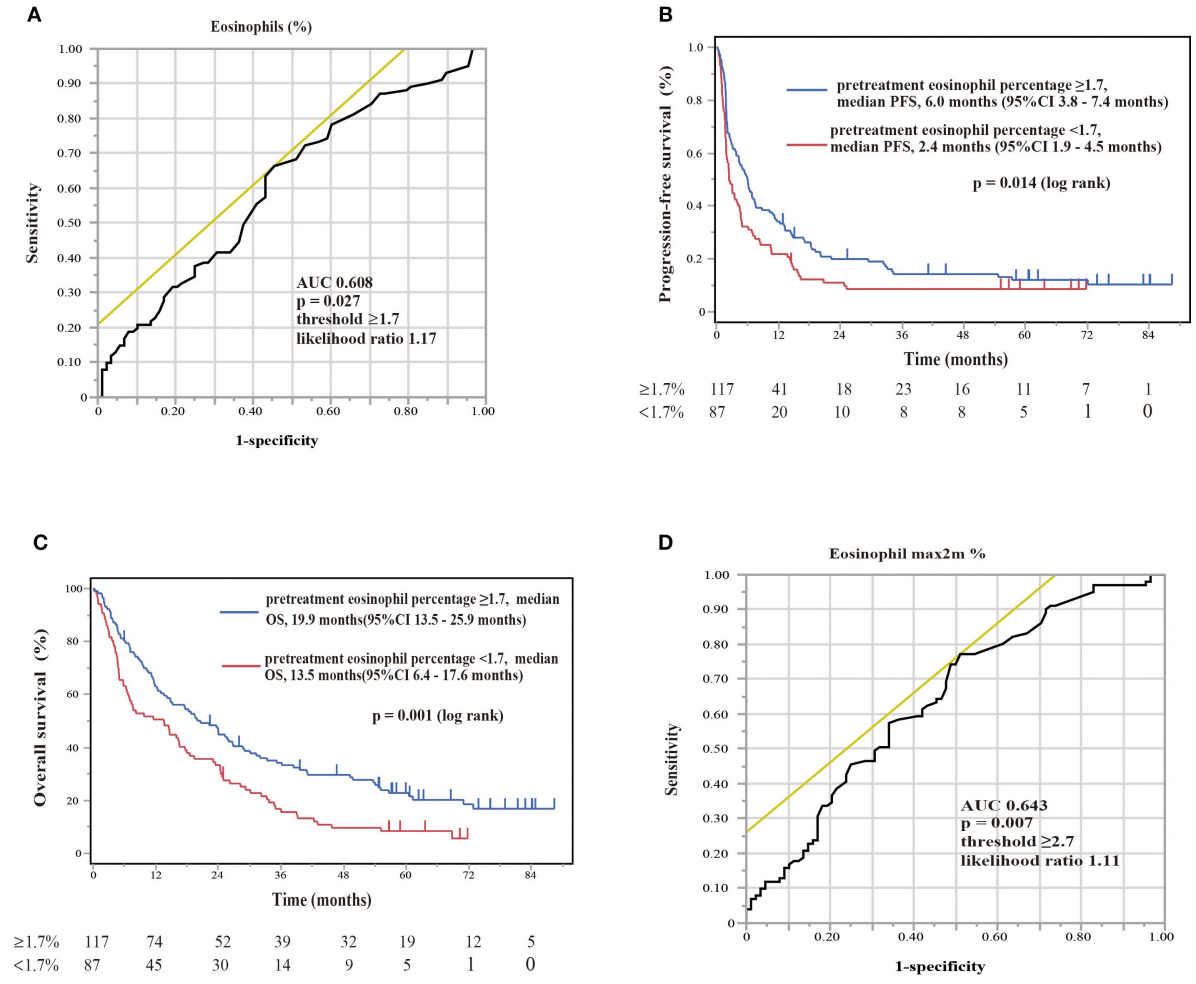
Diagnostic and prognostic evaluations of NSCLC patients treated with nivolumab monotherapy according to eosinophil percentage biomarker thresholds. **(A)** The ROC curve of the eosinophil percentage at the start of nivolumab assessing the diagnostic accuracy of a biomarker with an area under curve of 0.608 (optimal threshold ≥1.7%, sensitivity 66.3%, specificity 54.5%, likelihood ratio 1.17). The ROC curve provides a graphical representation of the trade-off between sensitivity and specificity for every possible cutoff value. **(B)** Kaplan–Meier plot for progression-free survival (PFS) stratified by eosinophil percentage. Patients with eosinophil percentages of ≥1.7% (pretreatment eosinophil percentage ≥1.7, n = 117) had a median PFS of 6.0 months (95%CI 3.8–7.4 months), whereas patients with levels of <1.7% (pretreatment eosinophil percentage <1.7, n = 87) had a median PFS of 2.4 months (95%CI 1.9–4.5 months). A log-rank test showed a significant difference (p = 0.014). Kaplan–Meier plots illustrate the probability of survival over time, with p-values indicating the significance of differences between groups. **(C)** Kaplan–Meier plot for overall survival (OS) stratified by the same eosinophil percentages. Patients with levels of ≥1.7% (pretreatment eosinophil percentage ≥1.7, n = 117) showed a median OS of 19.9 months (95%CI 13.5–25.9 months), in comparison to those with levels of <1.7% (pretreatment eosinophil percentage <1.7%, n = 87), with a median OS of 13.5 months (95%CI 6.4–17.6 months). A log-rank test showed a significant difference (p = 0.001). **(D)** The ROC curve of the maximum eosinophil percentage over 2 months (eosinophil max2m%) helps distinguish patients with controlled disease from those with progressive disease. The ROC curve for eosinophil max2m% indicates an AUC of 0.643, establishing the diagnostic relevance of this marker at a threshold of 2.7, with 77.2% sensitivity, 48.9% specificity, and a likelihood ratio of 1.11 (p = 0.007).

The therapeutic responses to nivolumab monotherapy in terms of eosinophil count and percentage at the start of nivolumab administration are shown in **Figure 6**. A therapeutic response was classified as a complete response (CR), partial response (PR), stable disease (SD), or PD, according to RECIST version 1.1. Patients who achieved a PR exhibited higher pretreatment blood eosinophil counts and percentages (**Figures 6A, B**). A substantial difference in pretreatment blood eosinophil percentage was observed between patients with a PR and those with either PD or SD (**Figure 6B**). Negative associations were observed between eosinophil count and eosinophil percentage and the change in tumor size after nivolumab monotherapy (eosinophil count, correlation coefficient = -0.28, p = 0.0004; eosinophil percentage, correlation coefficient = -0.29, p = 0.0002) (**Figures 6C, D**). An ROC curve analysis was used to distinguish between patients with controlled disease (CR + PR + SD) and those with PD. The goal was to pinpoint a specific cutoff value to efficiently separate the two groups based on disease status (area under the curve (AUC) = 0.608, threshold ≥ 1.7%, sensitivity = 66.3%, specificity = 54.5%, p = 0.027) (**Figure 7A**). Study participants were divided into eosinophilia and non-eosinophilia groups based on a cutoff value (determined by ROC curve analysis) of 1.7%, and PFS and OS were examined. There were no differences in the baseline characteristics, including age and sex, between the eosinophilia and non-eosinophilia groups, except for the presence of EGFR/ALK driver mutations (**Table 2**; p = 0.034). The PFS was significantly longer in the eosinophilia group than in the non-eosinophilia group (log-rank p = 0.014; **Figure 7B**). The eosinophilia group also showed a considerably longer OS than the non-eosinophilia group (log-rank p = 0.001; **Figure 7C**). In addition, we employed a similar ROC curve analysis using the eosinophil max2m% to discriminate between patients with controlled disease and PD. The AUC was 0.643 with a threshold of 2.7% (sensitivity, 77.2%; specificity, 48.9%; p = 0.007; **Figure 7D**). Furthermore, a higher eosinophil max2m% and a greater difference between eosinophil max2m% and pretreatment eosinophil percentage were both associated with prolonged PFS and OS (data not shown). We also conducted subgroup analyses stratified by treatment line. The prognostic value of baseline eosinophil percentage was significantly associated with improved PFS and OS in third-line or later therapy (p=0.0018 and p=0.0013, respectively) but not in second-line therapy. These findings further emphasize the potential of blood eosinophil level to serve as valuable indicators for predicting therapeutic responses and OS outcomes.

**Table 2:** Baseline data: patients with eosinophilia vs patients without eosinophilia.

Variables	Eosinophilia (≥1.7%, n = 117)	Non-eosinophilia (<1.7%, n = 87)	p-value
Age (median)	69(31-85)	70(38-80)	0.83
Sex Male/Female	88/29	60/27	0.34
PS 0-1/≥2	100/17	100/17	0.7
Smoking history Former/Never	96/21	62/25	0.089
Inhaled corticosteroids +/-	7/110	85/2	0.77
Allergic disease +/-	38/79	30/57	0.31
Histology Adeno/Squamous/Others	70/34/13	50/18/19	0.079
Disease stage III/IV/Recurrence	22/67/28	16/57/14	0.42
Mutation status Wild type/EGFR/ALK	102/15	64/22/1	0.034
PD-L1 (TPS) <1%/1-49%/≥50%/Unknown	14/11/3/89	10/8/2/67	0.99

PS, performance status; Adeno, adenocarcinoma; Squamous, squamous cell carcinoma; recurrence, recurrence after surgical resection; EGFR, epidermal growth factor receptor; ALK, anaplastic lymphoma kinase; PD-L1, programmed death ligand-1; TPS, tumor proportion score. Comparisons between the groups were performed using the chi-square test or Fisher’s exact test for categorical variables and the Mann–Whitney U test for continuous variables.

### 3.5 The role of blood eosinophilia in predicting the prognosis after nivolumab monotherapy in patients with and without EGFR/ALK driver mutations

**Figure 8:**
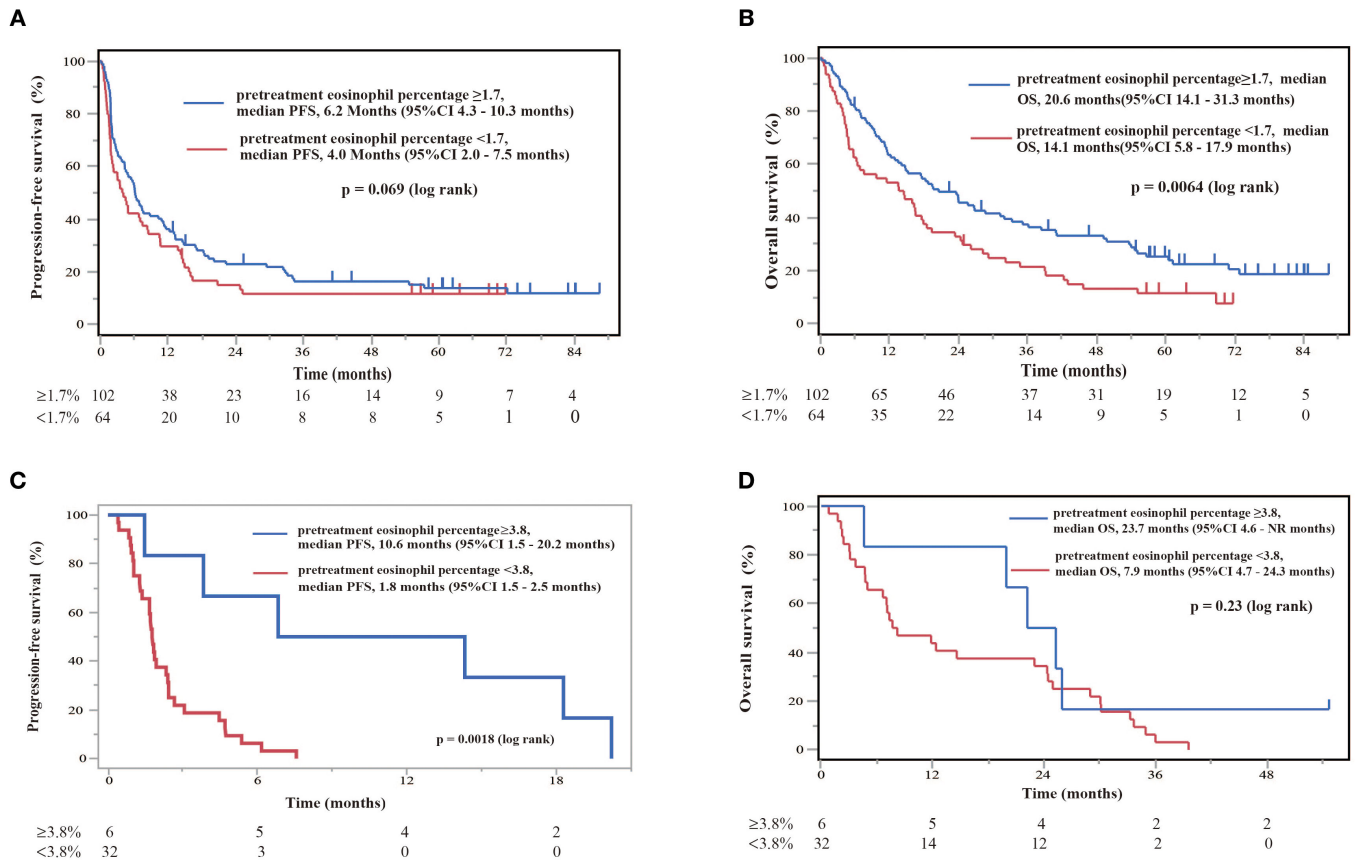
Diagnostic and prognostic evaluations of NSCLC patients treated with nivolumab monotherapy in the absence or presence of EGFR/ALK driver mutations according to eosinophil percentage thresholds. **(A)** Kaplan–Meier plot for PFS stratified by threshold eosinophil percentages in patients without EGFR/ALK driver mutations. Patients with eosinophil percentages of ≥1.7% (pretreatment eosinophil percentage ≥1.7, n = 102) had a median PFS of 6.2 months (95%CI 4.3–10.3 months), whereas patients with eosinophil percentages of <1.7% (pretreatment eosinophil percentage <1.7, n = 64) had a median PFS of 4.0 months (95%CI 2.0–7.5 months). Kaplan–Meier plots illustrate the probability of survival over time, with p-values indicating the significance of differences between groups. **(B)** Kaplan–Meier plot for OS stratified by the same eosinophil percentages in patients without EGFR/ALK driver mutations. Patients with levels of ≥1.7% (pretreatment eosinophil percentage ≥1.7, n = 102) showed superior median OS of 20.6 months (95%CI 14.1–31.3 months), in comparison to those with levels of <1.7% (pretreatment eosinophil percentage <1.7%, n = 64), with a median OS of 14.1 months (95%CI 5.8–17.9 months). A log-rank test showed a significant difference (p = 0.0064). **(C)** Kaplan–Meier survival curve illustrating PFS stratified by eosinophil percentage in patients with EGFR/ALK driver mutations. Patients with eosinophil percentages of ≥3.8% showed a median PFS of 10.6 months (95%CI 1.5–20.2 months), while those with levels of ≥3.8% had a median PFS of 1.8 months (95%CI 1.5–2.5 months). A log-rank test indicates a significant difference (p = 0.0018). **(D)** Kaplan–Meier plot for OS stratified by the same eosinophil percentages in patients with EGFR/ALK driver mutations. Patients with levels ≥3.8% showed a median OS of 23.7 months (95%CI 4.6– not reached (NR) months), in comparison to those with levels of <3.8%, who showed a median OS of 7.9 months (95%CI 4.7–24.3 months).

NSCLC patients with EGFR/ALK driver mutations do not respond well to ICI treatment. In this study, we analyzed 226 consecutive patients to reflect real-world clinical practice, including patients with driver mutations. Therefore, in our cohort, we performed an exploratory sub-analysis to examine the role of blood eosinophilia in predicting the prognosis after nivolumab monotherapy in patients with and without EGFR/ALK driver mutations (**Figure 8**). In patients without driver mutations, if the cutoff value for separating the eosinophilia group was set to 1.7%, OS was significantly longer in the eosinophilia group than in the non-eosinophilia group (log-rank p = 0.0064), although the prolongation of PFS was not significant (log-rank p = 0.069), probably because of the reduced sample size (**Figures 8A, B**). If the cutoff value was set to 1.7% in patients with EGFR/ALK driver mutations, PFS and OS did not differ between the eosinophilia and non-eosinophilia groups (data not shown). Therefore, we performed an ROC analysis based on the data of patients with EGFR/ALK driver mutations and found that the threshold of the eosinophil percentage was higher in patients with mutations than in all patients (eosinophil percentage: AUC = 0.79, threshold ≥ 3.8%, sensitivity = 71.4%, specificity = 96.6%, p = 0.0005). If this cutoff value (3.8%) was used, PFS, but not OS, was prolonged in the eosinophilia group of patients with EGFR/ALK driver mutations (PFS; log-rank p = 0.0018) (**Figures 8C, D**). A similar ROC curve was obtained when we analyzed the eosinophil max2m% in patients with EGFR/ALK driver mutations (eosinophil max2m%: AUC = 0.82, threshold ≥ 4.1%, sensitivity = 85.7%, specificity = 79.3%, p = 0.011). Moreover, a higher eosinophil max2m% and a greater difference between eosinophil max2m% and pretreatment eosinophil percentage were both associated with prolonged PFS in patients with driver mutations (data not shown). These findings suggest that, although the cutoff threshold is increased, blood eosinophil levels may have the potential to predict the prognosis after nivolumab monotherapy in patients with EGFR/ALK driver mutations.

## 4 Discussion

In this study, we found that a higher pretreatment blood eosinophil level or a greater subsequent increase in eosinophil count after nivolumab monotherapy was correlated with prolonged PFS and OS. Furthermore, blood eosinophil levels were correlated with prolonged PFS, even in patients with EGFR/ALK driver mutations, although the cutoff value was different from that of the overall cohort. Pretreatment blood eosinophil levels were positively correlated with the ratio of the EM subset in both CD4^+^ and CD8^+^ T cells, and that of ICOS cells in CD8^+^ T cells. Therefore, higher blood eosinophil levels may be associated with T cell activation and may be a promising biomarker for predicting a better response to nivolumab monotherapy in patients with NSCLC, regardless of the presence of driver mutations. Our findings contribute to the understanding of the potential role of eosinophils in cancer therapy, an area that has not been sufficiently explored.

PD-1 blockade therapy invigorates CD8^+^ T cells by inhibiting the binding of PD-L1 on antigen-presenting cells (APCs) and tumor cells to PD-1 on T cells, resulting in an antitumor effect (32–37). In contrast, CD4^+^ T cells have been shown to be required for priming CD8^+^ T cells, clonal proliferation, migratory invasive ability, and acquisition of the cell-killing function (33–36). It has been reported that the percentage of effector CD4^+^ T cells prior to treatment predicts the efficacy of PD-1 inhibitor therapy (35). The function of CD8^+^ T cells is thought to be mediated by Th1 cells, while Th2 cells, which have a seesaw balance with Th1 cells, have been thought to suppress anti-tumor activity (37).

Our results suggest that blood eosinophil levels are not associated with Th2 polarization or allergic diseases (**Figures 3** and **5**). Instead, the efficacy of nivolumab monotherapy in patients with high eosinophil levels appeared to be associated with the upregulation of EM subsets in CD4^+^ and CD8^+^ T cells (**Figure 4**). EM cells, a subset of memory T cells, circulate in peripheral tissues and the blood and can exert effector functions by producing large amounts of cytokines when they encounter antigens (38, 39). Several studies have shown that higher levels of EM T cells are associated with a better response to anti-PD-1 treatment and that anti-PD-1 treatment increases the levels of EM T cells (40–43). This corresponds to the finding that dendritic cells (DCs) express PD-L1 and that the PD-1/PD-L1 interaction plays an important role in DC-mediated T-cell activation. CD4^+^ and CD8^+^ T cells promote intratumoral infiltration by eosinophils (20, 44). The correlation between eosinophils, EM CD4^+^ T cells, and EM CD8^+^ T cells suggests that increased eosinophils may be due to increased EM CD4^+^ and CD8^+^ T cells induced by nivolumab monotherapy.

Blood eosinophil levels were positively correlated with the expression of ICOS in CD8^+^ T cells (**Figure 4**). ICOS is not expressed in naïve T cells and its expression increases with T cell activation. Similar to CD28, ICOS plays an important role in T-cell differentiation and activation. Furthermore, ICOS has been considered a candidate for gauging the therapeutic response to ICI treatment and as a biomarker for predicting the response to ICIs (e.g., nivolumab) (45, 46). In PBMCs, higher ICOS gene expression levels are associated with better outcomes (46). Moreover, eosinophils accumulate in the organs of T cell-specific roquin-deficient mice that overexpress ICOS (47), suggesting a potential role of ICOS in the accumulation of eosinophils. Overall, T-cell activation is associated with an increase in eosinophil levels and a better response to ICIs, including nivolumab. However, the correlation between blood eosinophil levels and EM subsets in CD4^+^, EM subsets in CD8^+^, and the ICOS expression in CD8^+^ T cells was modest (r < 0.5), suggesting that mechanisms other than T cell activation may have contributed to the increased level of blood eosinophils in this study.

In this study, we found that smoking history contributed to an increased pretreatment blood eosinophil count and increased eosinophil percentage (**Figures 3A, B**), which is consistent with previous studies (48). However, considering the intra-group differences in blood eosinophil levels in patients with a smoking history (**Figures 3A, B**) and that there was no difference in smoking history between the eosinophilia and non-eosinophilia groups (**Table 2**), the effect of smoking on peripheral eosinophil counts may not be linear, and may depend on the individual. Furthermore, smoking history was associated with longer PFS, but not OS, after nivolumab monotherapy (data not shown). Recent studies have suggested that ICI treatment is more effective in patients with a smoking history than in those without a smoking history (49, 50), which is consistent with our study. Therefore, it is possible that smoking increases blood eosinophil levels and thus improves the prognosis after nivolumab monotherapy. However, if the cutoff value for separating the eosinophilia group was set to 1.7% in patients with a smoking history, both PFS and OS were significantly longer in the eosinophilia group than in the non-eosinophilia group (data not shown), suggesting that blood eosinophil levels may be associated with the prognosis, independent of smoking history. Therefore, the relationship between smoking history and eosinophil counts in the context of tumor immunity after ICI treatment is complex and should be further investigated in the future.

The involvement of eosinophils in tumor immunity has not yet been fully elucidated. A positive correlation exists between the local tumor eosinophil count and blood eosinophil count (51). Tumor cells produce IL-5, GM-CSF, CCL11/eotaxin-1 (20, 21), all of which are involved in eosinophil activation and migration. However, type-1 cytokines/chemokines also activate eosinophils. Tumor cells not only produce IFN-γ and CXCR3 ligands but also release alarmin or damage-associated molecular patterns (DAMPs), which directly activate eosinophils, as previously reported (52–56). Furthermore, activated T cells, including EM cells, produce large amounts of cytokines, such as type-1 or type-2 cytokines/chemokines, which can activate and cause the migration of eosinophils into tumor cells, as described above. Therefore, several mechanisms are involved in the infiltration of eosinophils into tumors, including the production of cytokines/chemokines from tumor cells or memory T cells, and the release of DAMPs from tumor cells. However, the role of eosinophils in cancer development remains controversial (20–26). A protumoral role of eosinophils has been suggested in cervical cancer, lymphoma, and ovarian cancer (20, 21, 24–26). A pro-tumoral role of eosinophils has also been suggested in lung adenocarcinoma (21), probably through the production of pro-angiogenic factors, including vascular endothelial growth factor, and growth factors, including transforming growth factor-β. In contrast, an anti-tumor role of eosinophils has been suggested in colorectal, breast, and gastric cancer (20–23). This is probably due to the release of eosinophil-specific granules, such as MBP, and proteases, including granzyme B (20, 21, 57–60). Eosinophil lysate kills melanoma cells *in vitro* (57), and MBP has cytotoxic functions in several tumor cell lines (58). Moreover, eosinophils exert cytotoxicity by releasing proteases such as granzyme B (60). In addition, eosinophils directly adhere to tumor cells, which may play a role in tumor immunity. Therefore, there is a possibility that eosinophils can be activated by anti-PD-1 therapy and that activated eosinophils have a cytotoxic effect in controlling tumors. However, we did not examine the eosinophil function after PD-1 blockade therapy in this study.

It is also possible that eosinophils contribute to enhancing anti-tumor immunity by the direct activation of CD4^+^ and CD8^+^ T cells (61–66). Eosinophils have been reported to function as APCs, and airway eosinophils induce T-cell proliferation and cytokine production in T-cells (62, 63). Therefore, eosinophils may present tumor antigens to T cells for tumor-specific T cell activation. Furthermore, eosinophils produce and release cysLTs (64) and galectin-10 (Charcot-Leyden crystals) during eosinophil extracellular trap cell death (65), which directly activates DCs. In particular, DCs stimulated with galectin-10 activate not only Th2 cells but also Th1 cells (65) and thus can function in anti-tumor immunity. Moreover, anti-IL-5 treatment reduced the frequency of CD62L^-^CD45RA^-^ EM T cells and CD62L^+^CD45RA^-^ CM T cells (66) in patients undergoing asthma treatment. It also increases the frequency of CD62L^+^CD45RA^+^ naïve T cells and CD4^+^CD25^+^CD127^low^ Treg cells (66), suggesting that eosinophils can directly affect the ratio of CD4^+^ T cell subpopulations. Although the actual mechanism of eosinophil-related T cell activation has not been fully clarified, these findings suggest that eosinophils have anti-tumor activity not only through direct cytotoxic effects, but also through the activation of tumor-specific CD4^+^ and CD8^+^ T cells, which should be examined in the future.

The association between blood eosinophil counts and ICI efficacy has recently been highlighted (27–31). In patients with malignant lymphoma treated with ICI, some reports suggest that high pretreatment blood eosinophil counts or an early increase in eosinophil count after treatment are associated with an improved clinical response (27, 28). Furthermore, this association has also been explored in NSCLC, and similar findings have been suggested in some studies (29–31). Okauchi et al. analyzed 190 NSCLC patients treated with anti-PD-1 monotherapy (nivolumab or pembrolizumab) or the combination of anti-PD-1 treatment and chemotherapy and showed that the therapeutic effect was better when the eosinophil percentage exceeded 5% during treatment (30). Civil et al. analyzed 191 patients with NSCLC treated with anti-PD-1 or anti-PD-L1 monotherapy (pembrolizumab, nivolumab, atezolizumab, and durvalumab) and showed that the increase in eosinophil percentage during the treatment period was related to the efficacy and duration of treatment (31). These studies had either a small number of participants or combined miscellaneous treatments to increase the number of participants. Furthermore, evaluations of blood eosinophil counts and timing have been inconsistent among studies. Some studies used continuous variables, whereas others used categorical variables to evaluate blood eosinophil counts.

The present study has several limitations. This investigation was conducted at a single center, which may have resulted in bias. In addition, the male predominance (73%) may have introduced a sex-based bias in immune responses. Although we found a potential predictive role of eosinophil levels, even in EGFR/ALK-mutated NSCLC, the findings should be interpreted cautiously due to the limited sample size and baseline imbalances. We believe that multicenter studies with a more balanced sex or age distribution will be necessary to further clarify the relationship between eosinophils and tumor immunity and the effects of anti-PD-1 monotherapy. Second, we only nivolumab monotherapy in this study, and the relationship between blood eosinophil counts and clinical responses in patients treated with other ICIs was not investigated. Rather, the examination of a large number of patients (n = 204) using a single Ab (nivolumab) without chemotherapy (monotherapy) was considered a strength of this study. Third, all patients in our study had already received chemotherapy or other systemic treatments before starting nivolumab. Previous systemic therapies may have affected the circulatory immune environment, which make it difficult to apply our eosinophil findings to patients receiving immunotherapy as first-line treatment. Furthermore, as current practice often recommends chemo-immunotherapy for patients with PD-L1 <50% based on trials, such as KEYNOTE-189 (67), the predictive role of eosinophil levels in combination therapy remains unclear and warrants further study. Fourth, we did not investigate composite predictors that incorporate smoking status or EGFR/ALK mutations. Models that combine eosinophil counts with such characteristics may improve the predictive accuracy. Finally, we did not examine the functions of eosinophils, such as the generation of superoxide and the release of eosinophil granules, such as MBP, especially after the administration of anti-PD-1. An increase in blood eosinophil levels may reflect T cell activation. However, it is possible that eosinophils are activated by anti-PD-1 treatment, and that activated eosinophils can exert cytotoxic effects or induce cytotoxic T cell activation, as described above. We plan to examine the eosinophil function during anti-PD-1 therapy.

In conclusion, elevated eosinophil levels may indicate the activation of T-cell immunity but not the polarity of Th2-mediated responses in patients with NSCLC. Higher blood eosinophil levels before treatment or an early increase in eosinophil count after treatment suggest greater efficacy of anti-PD-1 therapy in NSCLC, at least in second-line or later treatment.

## Data Availability

The original contributions presented in the study are included in the article/**Supplementary Material**. Further inquiries can be directed to the corresponding authors.
