# Supplementary material for: Eosinophils as predictive biomarkers in anti-programmed cell death 1 monotherapy for non-small cell lung cancer
Source: Front Immunol. 2025 Oct 7;16:1574314. doi: 10.3389/fimmu.2025.1574314 (PMC12537673; doi:10.3389/fimmu.2025.1574314)
Supplement: Supplementary file 1 [file Table1.docx]

**Supplementary Materials**

**Eosinophils as Predictive Biomarkers in Anti-Programmed Cell Death-1 Monotherapy for Non-Small Cell Lung Cancer**

**Takahiro Uchida, Kazuyuki Nakagome, Kosuke Hashimoto, Hidetoshi Iemura, Yuki Shiko, Atsuto Mouri, Ou Yamaguchi, Yoshitaka Uchida, Yoshiaki Nagai, Tomoyuki Soma, Kyoichi Kaira, Makoto Nagata, and Hiroshi Kagamu**

**Supplementary Table 1.** Baseline data: Patients with PBMC analysis vs all patients

| Variables | PBMC  (n = 44) | All patients  (n = 204) | p-value |
| --- | --- | --- | --- |
| Age (median) | 69 (38-82) | 69 (31-89) | 0.58 |
| Sex  Male/Female | 30/14 | 148/56 | 0.56 |
| Line of therapy (≥2nd line)  2nd line/3rd line/ ≥4th line | 29/5/10 | 137/29/38 | 0.74 |
| PS  0-1/ ≥2 | 39/5 | 172/32 | 0.47 |
| Smoking history Former/Never | 34/10 | 158/46 | 0.98 |
| Inhaled corticosteroids  + / - | 3/43 | 9/195 | 0.60 |
| Allergic disease  + / - | 17/27 | 68/136 | 0.50 |
| Histology  Adeno/Squamous/Others | 25/13/6 | 120/52/32 | 0.84 |
| Disease stage  III/IV/Recurrence | 8/25/11 | 37/124/43 | 0.58 |
| Mutation status  Wild type/EGFR/ALK | 34/9/1 | 166/37/1 | 0.45 |
| PD-L1 (TPS)  <1%/1-49%/ ≥50%/Unknown | 5/6/3/30 | 24/19/5/156 | 0.37 |

PS, performance status; Adeno, adenocarcinoma; Squamous, squamous cell carcinoma; recurrence, recurrence after surgical resection; EGFR, epidermal growth factor receptor; ALK, anaplastic lymphoma kinase; PD-L1, programmed death ligand-1; TPS, tumor proportion score. Comparisons between the groups were performed using the chi-square test or Fisher’s exact test for categorical variables and the Mann–Whitney U test for continuous variables.
